# Current challenges and controversies in the management of scapular fractures: a review

**DOI:** 10.1186/s13037-020-00281-3

**Published:** 2021-01-06

**Authors:** Robinson Esteves Pires, Vincenzo Giordano, Felipe Serrão Mendes de Souza, Pedro José Labronici

**Affiliations:** 1 Departamento do Aparelho Locomotor, Universidade Federal de Minas Gerais, Av. Prof. Alfredo Balena, 190, Santa Efigênia, Belo Horizonte, MG Brazil; 2 Serviço de Ortopedia e Traumatologia, Instituto Orizonti, Belo Horizonte, MG Brazil; 3 Serviço de Ortopedia e Traumatologia Professor Nova Monteiro, Rio de Janeiro, RJ Brazil; 4 Clínica São Vicente, Rede D’Or São Luiz, Rio de Janeiro, RJ Brazil; 5 Serviço de Ortopedia e Traumatologia, Hospital Santa Teresa, Petrópolis, RJ Brazil

**Keywords:** Scapular fractures, Surgical decision-making, Non-operative treatment, Floating shoulder, Complications, Patient safety

## Abstract

Fractures of the scapula are rare and usually associated with high-energy trauma. The unfavorable scapular anatomy, combined with the complexity of the approaches for fracture fixation, make the treatment challenging, even for experienced surgeons. Furthermore, the literature is controversial regarding surgical indications and rationale for treatment. The present review article was designed to address and discuss critical aspects of decision-making for the management of scapular fractures, including surgical indications and patient safety considerations.

## Background

Direct impact may cause fractures in all scapular regions, but an indirect trauma caused by the humeral head into the glenoid fossa also may cause intra- and extra-articular scapular fractures [1–4].

Although radiographs are essential for diagnosis, including anteroposterior, lateral, and axillary views, computed tomography (CT) plays a critical role in the preoperative planning and decision-making process, especially three-dimensional (3D) reconstructions [4]. Armitage et al. [5] mapped 90 scapula fractures using 3D-CT and found that 68% involved the inferior aspect of the scapula neck. Seventeen percent of fractures presented articular involvement, while 84% traversed medially to exit inferior to the medial extent of the scapular spine. However, the authors highlighted that articular fractures did not follow a predictable pattern.

Scapular fractures may significantly impair the normal function of the shoulder girdle, causing chronic pain as a result of impingement, malunion, nonunion, or scapulothoracic dyskinesis [4].

In the herein review, we present an overview of all scapula fracture types, focusing in the treatment strategy for safe management of the most frequent and important fracture patterns.

### Classification systems for scapular fractures

Several classification systems have been described for scapula fractures, according to the pattern, number of fragments, location, and prognosis. The most important and universally used classification systems for scapula fractures will be detailed below.

The first description of a classification system for fractures of the scapular body is credited to Petit, in 1723, who divided the body fractures into three patterns, according to the orientation of the fracture line: transverse, oblique, and longitudinal [6].

Ada and Miller [7] described a classification system based on a retrospective experience of 116 scapulae. The authors named fractures of the acromion and coracoid process in types I and II, respectively. Three types of neck fractures were also described, according to the course of fracture lines: Type IIA (fractures of the surgical neck); type IIB (transpinous scapular neck fractures); and type IIC (transverse fractures of the scapular body). Later, Goss [8] modified the Ada and Miller [7] classification system, excluding transpinous scapular neck fractures, and including fractures of the anatomical neck. The author named the IIC type a fracture of neck inferior to scapula spine.

Hardegger [9–11] classification is quite similar to the Ada and Miller system and names two types of neck and two types of glenoid fractures.

The revisited AO/OTA classification system for scapula fractures is codified as following: 14 (scapula bone); A (acromion or coracoid process); B (body), and F (glenoid fossa). Qualificators should be included according to the fracture location [12].

Bartoníček et al. [10] described an interesting classification system for fractures of scapula body based on the findings of 187 CT scans of patients presenting fractures in this location. The authors divided the scapular body fractures into three major groups: fractures of the spinal pillar; fractures of the lateral pillar (subtypes: Two-part, three-part, and comminuted fractures); and fractures of both pillars (subtypes: Fractures involving the medial third of the spinal pillar and fractures involving the central part of the spinal pillar).

The Ideberg et al. [13] classification is the most accepted system for glenoid cavity fractures of the scapula. The authors grouped glenoid fractures based on a series of 338 patients. In summary, this classification that received later modifications by Goss et al. [8] and Mayo et al. [14] divides the fracture patterns into glenoid rim fractures (type I) and glenoid fossa fracture with increasing degrees of scapular neck and body involvement (types II-VI).

Several classification systems have been described for coracoid process fractures, including Tanton [15], Eyres [16], Ogawa [17], Goss [8], and AO-OTA [12]. The Bartoníček [15] classification system, based on the fracture location and presence of comminution, is divided into four groups: Type I (fracture of apex); Type II (fracture of beak); Type III (fracture of base); Type IV (comminuted fracture).

The same classification systems above mentioned also address acromion fractures [8, 12, 17–19].

Kuhn et al. [19] described the acromion fractures divided into three categories: Type I (fractures can be with or without a slight dislocation. Subtype IA depicts avulsion and subtype IB, true fracture); Type II (fractures are dislocated but without constraint in the subacromial space); Type III fractures are dislocated and constrain the subacromial space [19].

Even knowing that there is no ideal classification for scapula fractures, our preferred systems are Bartoníček et al. [10] (body fractures), Ideberg et al. [13] (glenoid fossa and rim fractures), Bartoníček et al. [15] (coracoid process fractures), and Kuhn [18] (acromion fractures).

### Treatment strategies for scapula fractures

#### Conservative treatment for scapular fractures

The vast majority of scapula fractures (> 80%) are amenable to conservative treatment and present favorable functional outcomes [19–21]. In this scenario, the majority of the isolated scapular body and glenoid neck fractures, as well as almost all acromion, coracoid process, and scapular spine fractures are adequately managed nonsurgically [22]. In a systematic review of 520 scapula fractures, Zlowodzki et al. [22] found that 99% of all isolated scapula body fractures and 83% of all glenoid neck fractures were treated nonoperatively, with excellent or good results achieved in up to 86 and 77% of the cases, respectively. Conversely, these authors observed that 80% of all glenoid fossa fractures were managed operatively, with excellent or good results in 82% of the cases.

Conservative treatment consists initially of pain control and immobilization with a sling, followed by physical therapy. Passive-assisted exercises start after pain control (usually after 14 days). Active-assisted exercises usually start after 21 days, according to the patient tolerance. Active exercises are usually initiated after 28 days.

Schofer et al. [23], in a retrospective cohort study of 51 patients with an average follow-up of 65 months, showed good functional outcomes after conservative treatment of scapula fractures.

#### Surgical indications

The treatment of scapula fractures has been changing substantially in the last decade. Although the scapula has a privileged muscular envelope which uneventfully heals the great majority of fractures, a scapular malunion may significantly impair the shoulder girdle function, causing chronic pain, aesthetic deformities, impingement, and scapulothoracic dyskinesis.

The literature is extremely controversial regarding the surgical indications for scapula fractures. Several studies pointed out indicators for surgical management, but we are currently quite far from consensus [11, 14, 20].

Besides patient characteristics such as age, arm dominance, previous function, and type of occupation, the relative operative indications are presented below:

- Articular displacement or gap > 4 mm;
- Articular involvement > 20 to 25%;
- Medialization of the scapula > 20 mm (reduced to 10 mm for double disruptions and 15 mm when combined with 30^o^ angulation);
- Glenopolar (GP) angle ≤22°;
- Angulation ≥45°

*Source: The Scapula Institute – St. Paul / Minnesota (**www.scapulainstitute.org**).*

Careful evaluation of the GP angle should be performed to prevent misinterpretation of the correct measurement. A GP angle ranging from 30° to 45° is considered normal^20^. However, Labronici et al. [20] recommended that, whenever possible, measurement of GP angle should be taken in neutral rotation, since rotation of the scapula can either increase or decrease the measurement, therefore leading to a possible non-ideal indication for surgery.

Kim et al. [21] showed a positive relationship between smaller GP angle and poor Constant-Murley functional outcome in floating shoulders.

#### Fractures of the glenoid neck and body of the scapula

Fractures of the glenoid neck and body usually result from high-energy trauma and a high degree of suspicion of associated injuries must be observed [24]. Depending on patient characteristics, such as age, arm dominance, degree of previous functional activity, as well as in the presence of the above mentioned anatomical indications, surgical treatment should be beneficial to achieve favorable outcomes.

Tatro et al. [25], in a case series of 66 patients who underwent open reduction and internal fixation (ORIF) for treatment of scapular fractures (37 extra- and 29 intra-articular patterns), showed excellent functional outcomes after long-term follow-up, ranging from 5 to 10 years. Interestingly, these authors reported comparable outcomes after intra- and extra-articular fractures.

For approaching both glenoid neck and scapular body fractures, we place the patient in lateral decubitus with a contralateral axillary roll and the chest slightly anteriorly positioned. Alternatively, the patient can be positioned in ventral decubitus if there is no associated chest injury or pulmonary contusion. The ipsilateral arm is properly draped and placed in 90^o^ with the chest, freely resting over a pillow. The C-arm is positioned over the patient.

The choice of approach and fixation strategy depend on the fracture location, number of fragments, and degree of displacement [26].

**Fig. 1:**
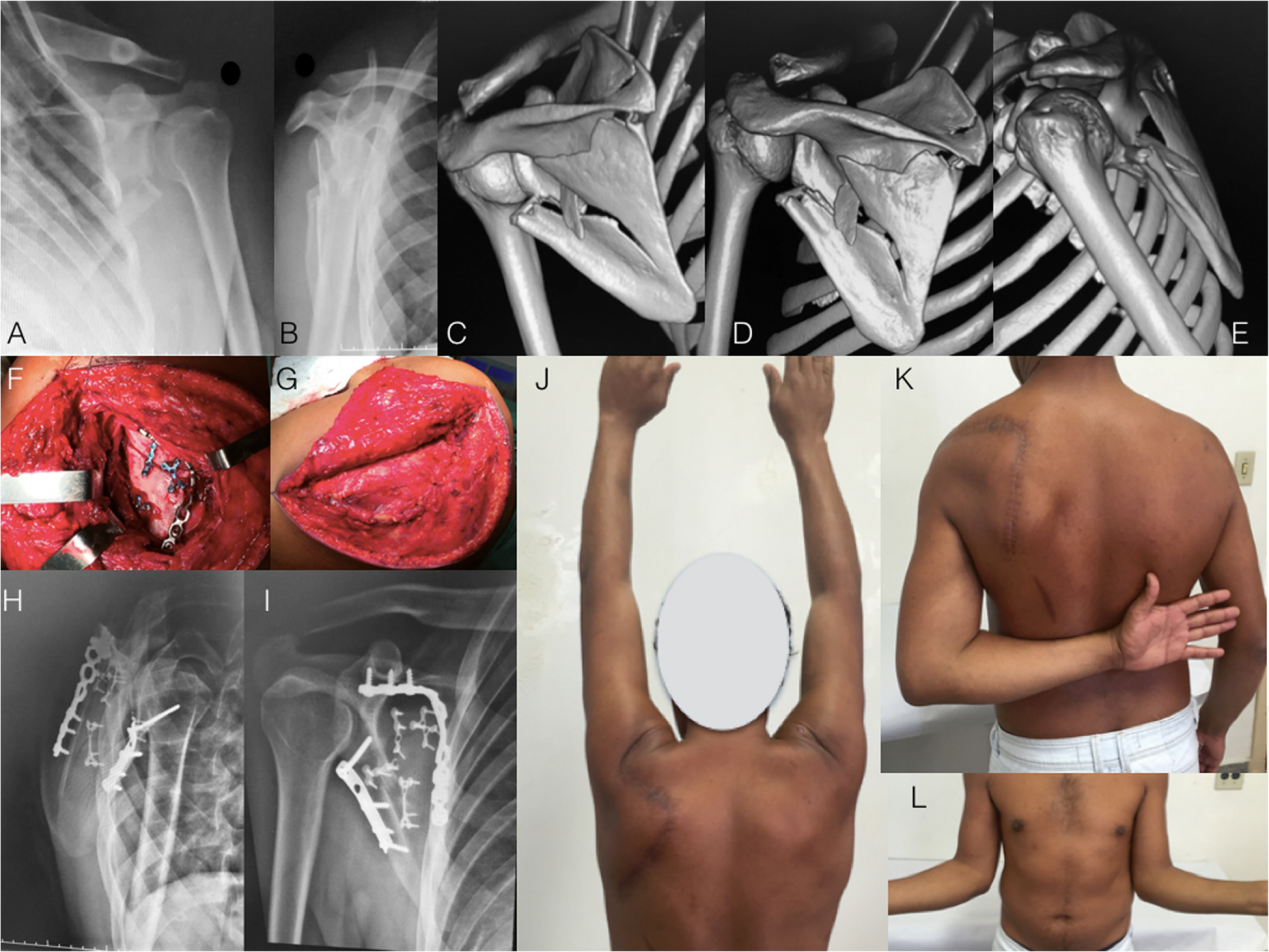
**a** and **b**: Radiographs of the left shoulder of a 34-year-old male patient who suffered a motorcycle accident and presented a severely displaced and comminuted infraspinous fracture of the scapula body. **c**, **d**, and **e**: Computed tomography with three-dimensional reconstruction. Observe the angulation of the scapula body and the degree of glenoid medialization. **f**: Perioperative photography depicting the classic Judet approach. Observe the extensile detachment of the infraspinatus muscle. **g**: Perioperative photography showing the closure and infraspinatus muscle. **h** and **i**: Radiographs after three months showing the fracture healing after fixation with one-third tubular plate at the lateral pillar and a twisted reconstruction plate at the medial pillar of the scapula. Fragment-specific fixation using 2.0-mm minifragment plates was also performed. **j**, **k**, and **l**: Postoperative photographs showing complete range of motion recovery after three months of surgery

The classic approach for scapula fracture fixation was described by Judet [27]. Although universally accepted as a helpful and effective approach, especially for complex fracture patterns and delayed fixations, the classic Judet involves extensile dissection of the infraspinatus muscle, which negatively impacts the rehabilitation process and increases the risk of iatrogenic damage to the suprascapular nerve due to prolonged retraction. Moreover, a postoperative seroma is a relatively frequent complication that usually requires drainage. In our practice, we currently reserve the Judet approach for delayed fractures and malunions. Figure 1 shows a case of patient who underwent open reduction and internal fixation of the scapula after 21 days of trauma. The indication for surgical treatment was based on the medialization of the glenoid (> 20 mm) and the GP angle (20^o^).

**Fig. 2:**
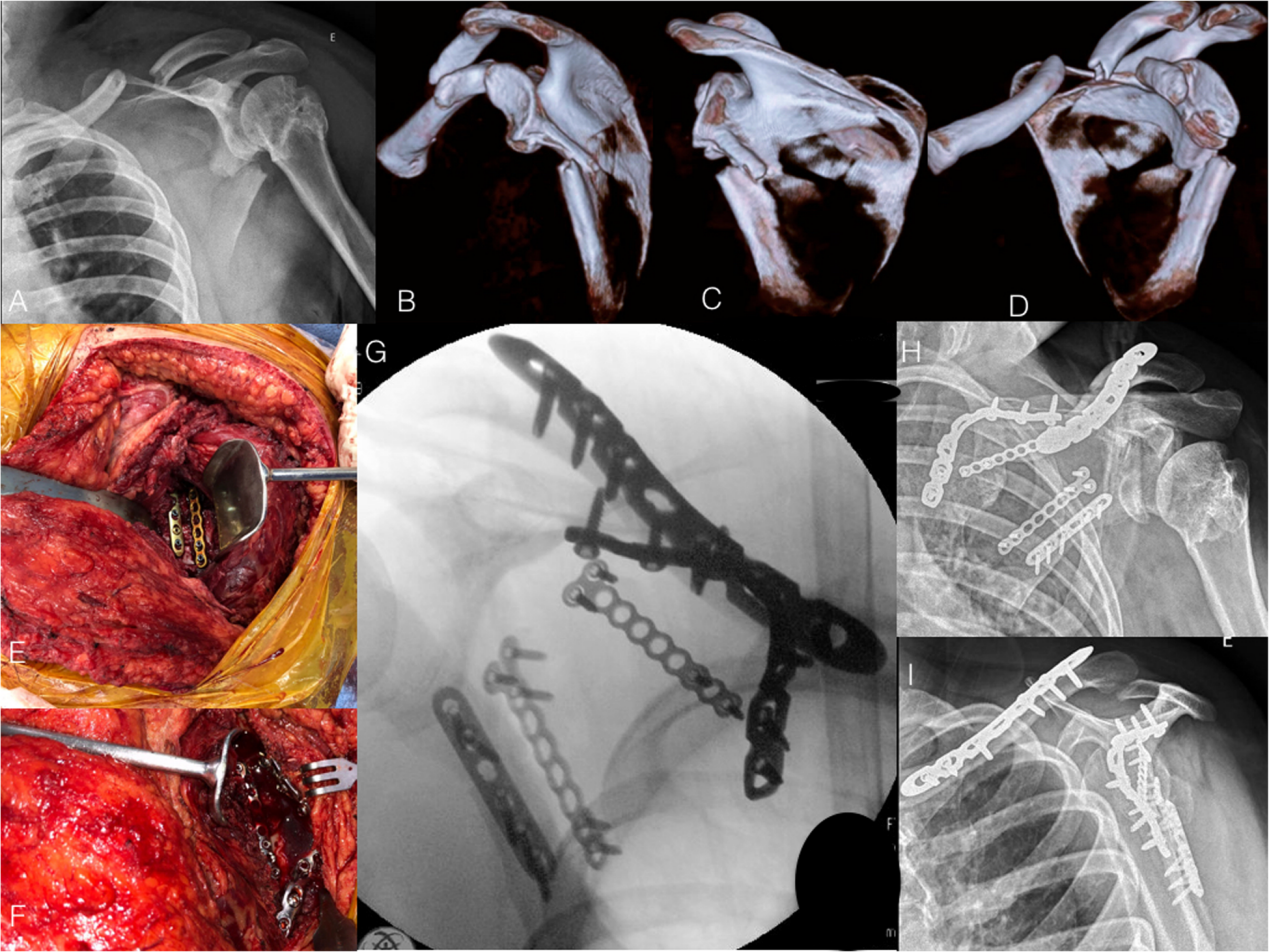
**a**: Radiograph of the right shoulder in anteroposterior view of a 24-year-old male patient who suffered a car accident and presented a severely displaced midshaft clavicle fracture in combination with an infraglenoid fracture of the scapula body. Observe that the patient presented a sequelae of previous proximal humeral and glenoid fractures, with no residual shoulder instability. **b**, **c**, and **d**: 3-D CT reconstruction showing the medialization of the glenoid and the angulation of the scapular body. **e** and **f**: Perioperative photographs depicting the modified Judet approach. Observe the fixation of the lateral pillar of the scapula with two plates at the interval between the infraspinatus and teres minor muscles (**e**). The medial pillar of the scapula was reduced and fixed with a twisted reconstruction locking plate. Observe the minimal detachment of the infraspinatus muscle (**f**). **g**: Perioperative fluoroscopy image showing scapula and clavicle fractures reduction and fixation. **h** and **i**: Radiographs in anteroposterior and lateral views showing fracture healing after three months

Further, Obremskey and Lyman [28] described the modified Judet approach, using the same skin incision (so-called boomerang incision), but preserving the infraspinatus attachments. The authors advocate approaching the lateral pillar of the scapula using the interval between the infraspinatus and the teres minor. If the medial pillar of the scapula must be addressed, partial detachment of the infraspinatus should be carefully performed (Fig. 2). Intraoperatively, the scapular circumflex artery should be found and ligated, as an inadvertent damage to this structure during dissection in the infraspinatous-teres minor interval causes a persistent bleeding and increases the surgical time. Advantages of this modified approach include less risk of neurological damage, less bleeding, and better shoulder function. Nevertheless, the large skin incision is still a major cosmetic concern with the modified Judet approach.

Salassa et al. [29], in a cadaveric study, showed that the modified Judet approach without posterior deltoid takedown allows for safe exposure of the lateral pillar of the scapula and direct visualization of the critical neurovascular bundle. The authors recommend beginning the exposure with the posterior deltoid origin left intact and only proceed with takedown if additional exposure is needed, usually in complex fracture patterns.

Although both classic and modified Judet approaches are considered safe and well-stablished treatment options for scapular fixation, caution must be taken to avoid neurovascular damage when developing the intermuscular dissection to access the lateral pillar of the scapula. Costa et al. [30], in a cadaveric study, found a mean distance from the infraglenoid tubercle to the axillary nerve of 23.8-mm, and to the suprascapular nerve of 33.2-mm.

A straight simplified longitudinal approach described by Brodsky [31] is also possible, especially for fracture patterns when fixation of the medial pillar is not required. We believe that this approach is an interesting alternative for fractures of the lateral pillar of the scapula in association with displaced acromion fractures. In such cases, a proximal extension of the standard longitudinal straight approach is performed to allow for proper acromion fixation. Also, the posterior glenohumeral capsule can be opened to allow for better articular visualization, when there is an associated articular fracture line to the glenoid fossa.

**Fig. 3:**
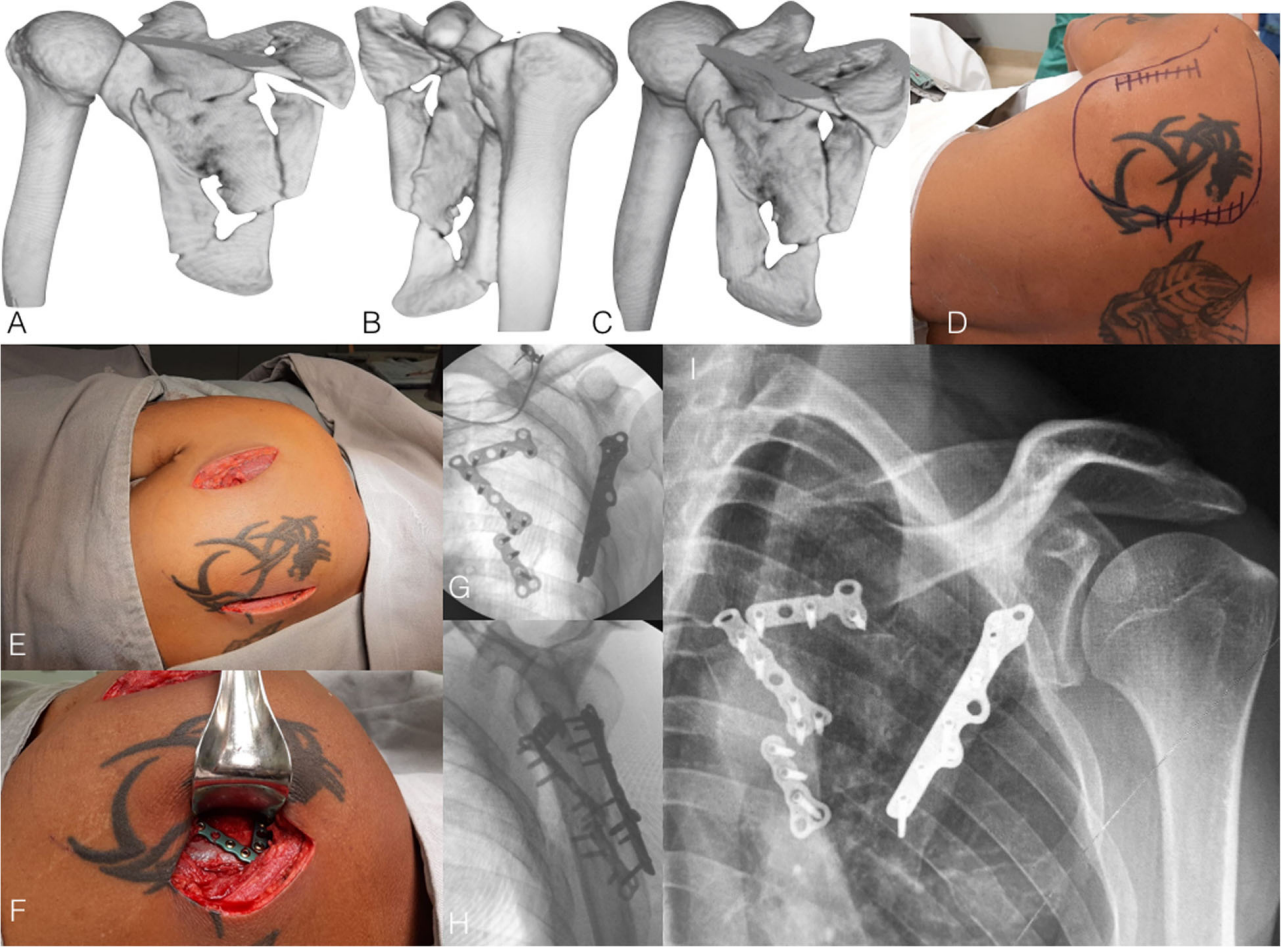
**a**, **b**, and **c**: 3-D CT reconstruction showing a comminuted infraglenoid fracture of the scapular body in a 35-year-old male patient. Observe the angulation of the inferior part of the scapular body and the medialization of the glenoid. **d**: Preoperative photography depicting the landmarks for minimally invasive approach. **e** and **f**: Perioperative photographs showing the lateral (between infraspinatus and teres minor muscles) and medial approaches (partial detachment of the infraspinatus). **g** and **h**: Postoperative fluoroscopy images in anteroposterior and lateral views showing fracture reduction and fixation using 2.7 minifragment plates (medial pillar) and the unconventional use of a 2.7 fibular plate (lateral pillar)

Gauger and Cole [32] described a minimally invasive approach to scapula neck and body fractures where incisions are made along the scapula borders for reduction and fixation. In a case series of seven patients with a minimum follow-up of 12 months, the authors highlighted that this novel technique allows adequate visualization of fracture reduction without extensile muscular or subcutaneous flaps and was associated with satisfactory functional outcomes (Fig. 3).

**Fig. 4:**
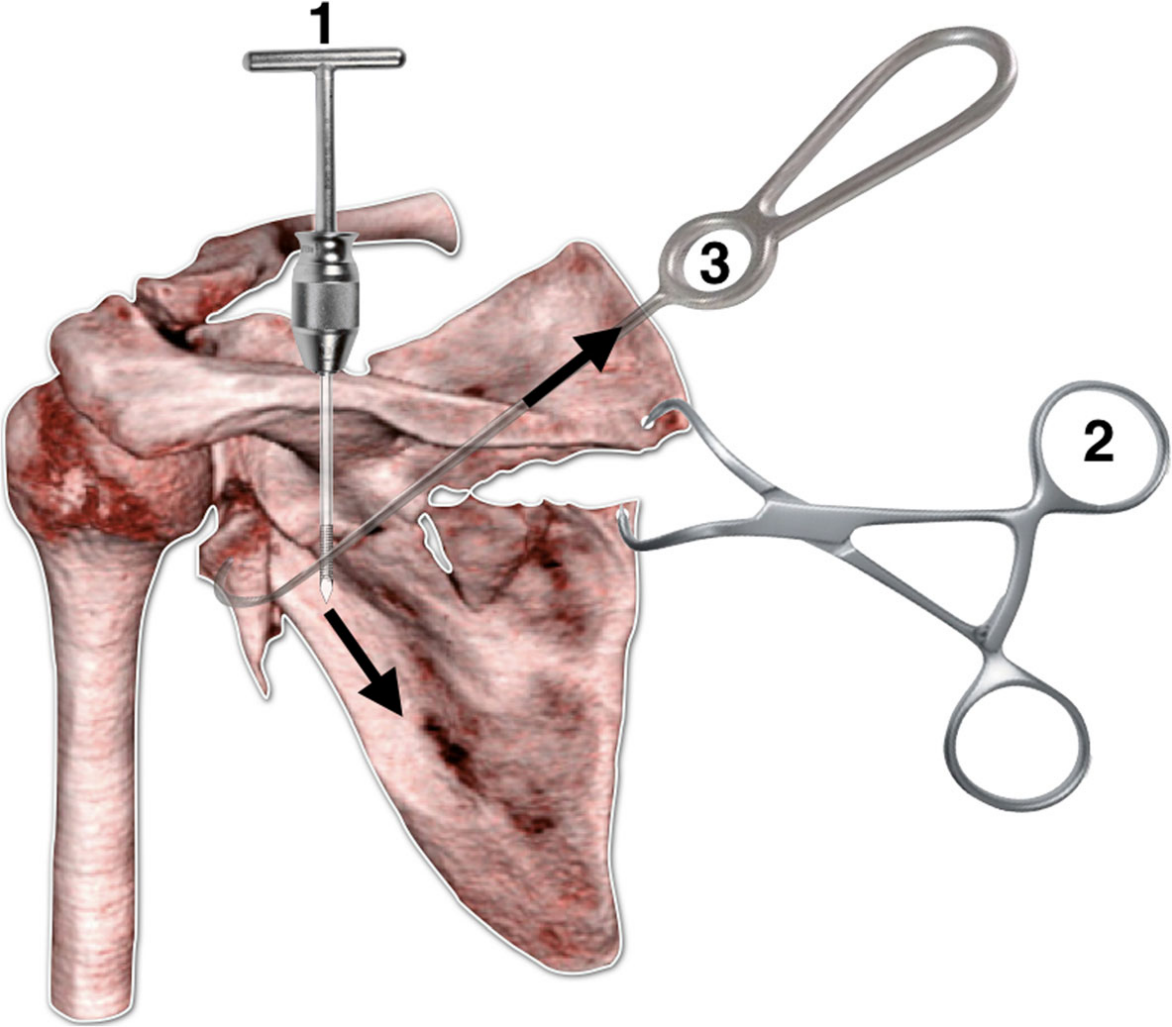
Illustration simulating the sequence of reduction of a displaced fracture of the glenoid and body of the scapula. The reduction starts with the placement of a Schanz screw in the body of the scapula and a traction in the caudal direction is performed to correct the length of the lateral pillar. Then, two holes are performed with a 2.5-mm drill bit on each side of the medial pillar of the scapula and a pointed clamp is used for medial column reduction. Following, a bone hook is used to pull the glenoid fragment in order to achieve reduction. Provisional K-wires or miniplates may be used for reduction maintenance

The decision-making on where to start the fracture reduction (medial or lateral pillar) depends on the fracture pattern. If the fracture is amenable for fixation of just one pillar, we recommend starting the reduction on the most displaced column (usually, the lateral). If both pillars are severely displaced, we generally perform a modified Judet or a minimally invasive approach simultaneously addressing both pillars to adequately manipulate the fracture fragments. Nevertheless, generally the medial pillar has to be reduced and fixed with a relatively flexible implant first as it acts as a hinge to allow better manipulation, reduction, and final fixation of the lateral pillar. Reduction instruments such as pointed reduction clamps, bone hook, and small-diameter Schanz pins with a T-handle are essential to obtain satisfactory reduction (Fig. 4).

An important issue regarding scapula fractures lies on the complex and unfavorable anatomy of the scapula for proper fixation. Specially-designed pre-contoured implants are not universally available. Therefore, the surgeon is frequently obligated to use implants that were not specifically designed for scapula fractures. Hu et al. [33], in a retrospective cohort of 37 patients, reported favorable functional outcomes using distal humeral Y-type locking plates. No plate rupture and screw prolapse were observed during the 1-year follow-up.

Our fixation strategy usually combines stronger constructs (3.5- or 2.8-mm locking and non-locking compression and/or reconstruction plates, with 2.4- or 2.0-mm minifragment plates), in a fragment-specific fixation to achieve a stable construct [34].

Our postoperative care includes pain control and sling for two weeks. Passive exercises are allowed after the first week. Active-assisted exercises are initiated after two weeks. Active exercises are allowed after three weeks and progressive strengthening is started after six weeks.

#### Floating shoulder

The definition of floating shoulder remains controversial. Some authors described the floating shoulder when two or more structures of the superior shoulder suspensory complex (SSSC) were disrupted [35–37].

Bartoníček et al. [38] postulate that floating shoulder should be considered an unstable displaced fracture of the anatomical or surgical glenoid neck of the scapula in association or not with a clavicle fracture. The authors highlighted that, in cases of surgical neck fractures, there must occur an associated rupture of both the coracoacromial and coracoclavicular ligaments or a fracture of their osseous-equivalent structures (extra-articular or intra- or extra-articular coracoid base and acromion). The combination of a midshaft clavicle fracture with a scapular body fracture is frequently misinterpreted as a floating shoulder. This injury pattern has no influence on stability or displacement of the glenoid neck. Consequently, the only fixation of the clavicle usually does not result in improvement of the displacement of the scapula [38].

Although some degree of improvement of the GP angle compared pre- and postoperatively was reported after fixation only of the clavicle, we do not routinely observe such improvement, which we believe can be attributed to associated capsuloligamentous injuries of the SSSC [21].

The treatment of floating shoulder also remains a topic of debate. While some authors advocate conservative treatment, others defend fixation of the clavicle alone and, a third group, fixation of both, clavicle and scapula [39–41].

Cunningham et al. [42], in a case series of 41 patients presenting association of floating shoulder and flail chest, compared 23 treated with operative stabilization and 18 treated non-operatively. The authors found that restoration of the scapula-clavicular arch unloads of the flail chest and may improve respiratory function and pain control, thereby decreasing duration of mechanical ventilation days and intensive care unit length of stay.

Our treatment protocol for floating shoulder is the fixation of the clavicle alone, if the scapular neck presents no displacement or minimal displacement and the 3-D CT reconstruction GP angle in neutral rotation is >22^o^. Otherwise, we fix both, clavicle and scapula, starting fixation with the clavicle, in a beach chair position. After clavicle fixation, we place the patient lateral to perform scapula fixation, either using the modified Judet approach or preferably a combination of minimal approaches. All efforts are made to preserve the posterior deltoid insertion. We currently also consider restoration of the scapula-clavicular arch, fixing both scapula and clavicle in patients with floating shoulder and flail chest. The postoperative care is the same previously described for glenoid neck and body scapula fractures.

#### Fractures of the glenoid fossa and rim

Displaced glenoid fossa and rim fractures are caused by direct high-impact lateral trauma and are preferably managed by open reduction and internal fixation due to a high-risk of chronic instability of the shoulder and degenerative joint disease [43]. Associated skeletal and non-skeletal injuries, mainly in the thoracic region, are common and sometimes life-threatening^43,44^. The most common classification system for glenoid cavity fractures of the scapula was described by Ideberg et al. [13], later modified by Goss et al. [8] and Mayo et al. [14]. In this classification system, fractures are separated into glenoid rim-type fractures (types IA and IB) and glenoid fossa fractures (types II-VI). True fractures of the glenoid rim are distinct from the small avulsion-type fractures, generally seen after a dislocation of the humeral head, such as the so-called false Bankart osseous lesion [43, 44]. Conversely, true fractures of the glenoid rim are generally larger and occur when a lateral force drives the humeral head directly against this structure with or without a shoulder dislocation [43, 44]. Type-IA represents an anterior rim fracture and type-IIB represents a posterior rim fracture of the glenoid cavity [13].

Glenoid fossa fractures occur after a violent force applied laterally to the proximal part of the humerus, which is driven into the glenoid cavity. Most articular fractures involve only part of the glenoid fossa, with the intact portion of the articular surface remaining in normal anatomical relationship with the scapular neck or scapular body [45]. A transverse fracture line traverses the glenoid cavity and propagates in several directions, dictating different fracture patterns [13, 44]. In type-II fractures, the force is directed somewhat inferiorly, with the fracture line running to the lateral border of the scapular body and creating a displaced inferior fragment. In type-III fractures, the force is directed somewhat superiorly, with the fracture line exiting along the superior border of the scapula and sometimes disrupting some structures of the SSSC. The fracture fragment includes the coracoid process and the superior articular surface of the glenoid cavity. In type-IV fracture the force is driven centrally, and the fracture line runs across the scapula, exiting along its medial border. The scapula is split transversely into a smaller superior fragment and a larger inferior fragment. Type-V fracture is a combination of types II, III, and IV fracture patterns, presenting three variants. In type-Va variant, the main fracture line runs across the scapula, exiting along its medial border, and a secondary fracture line runs to the lateral border of the scapula, creating a separate inferior fragment. In type-Vb variant the main fracture line runs across the scapula, exiting along its medial border, and a secondary fracture line runs to the superior scapular margin, creating a separate superior fragment. In type-Vc variant, the main fracture line runs across the scapula, exiting along its medial border, and a secondary fracture line runs to both the superior and the lateral borders of the scapular body, creating separate superior and inferior glenoid fragments. Type-VI fracture is a severely comminuted injury affecting the entire glenoid fossa and is termed total glenoid fracture [45]. Despite its detailed anatomical characterization, the modified Ideberg et al. classification [8, 13, 14] has some limitations imposed mainly by the use of standard radiographs only and its purely descriptive nature, with little or no therapeutic or prognostic applicability [46].

Using 3D CT reconstructions and intraoperative findings, Bartoníček et al. [46] developed a classification system of glenoid fractures with five basic types of injuries identified based on analysis of separated portion of the glenoid fossa. Basic types of glenoid fossa fractures include fractures of the superior glenoid, the anterior glenoid, the posterior rim of the glenoid, the inferior glenoid, and the entire glenoid (total glenoid fracture), which are dictated mainly by the direction of the deforming force and the position of the arm at the moment of the traumatic injury. The superior glenoid fracture involves the upper part of the glenoid fossa and extends as far as the upper border of the scapula, with the fracture line propagating to the supraspinous fossa (above the spinal pillar of the scapula). The anterior glenoid fracture is characterized by a separation of the anterior and sometimes part of the lower glenoid fossa rim. The posterior glenoid fracture involves avulsion of the posterior rim of the glenoid fossa, which can extend as far as its lower rim. Comminution is relatively common in this fracture pattern, with smaller fragments remaining together by the glenoid labrum. The inferior glenoid fracture is characterized by a separation of the distal fragment of the glenoid fossa, with varying extension into the lateral border of the scapula body. In the entire glenoid fracture, all parts of the articular surface are separated from the scapular neck or body. Bartoníček et al. [46] identified four cases of entire glenoid fracture combined with comminuted fractures of the whole scapular body. We call this combination a complex fracture of the scapula, which will be addressed later in this review.

**Fig. 5:**
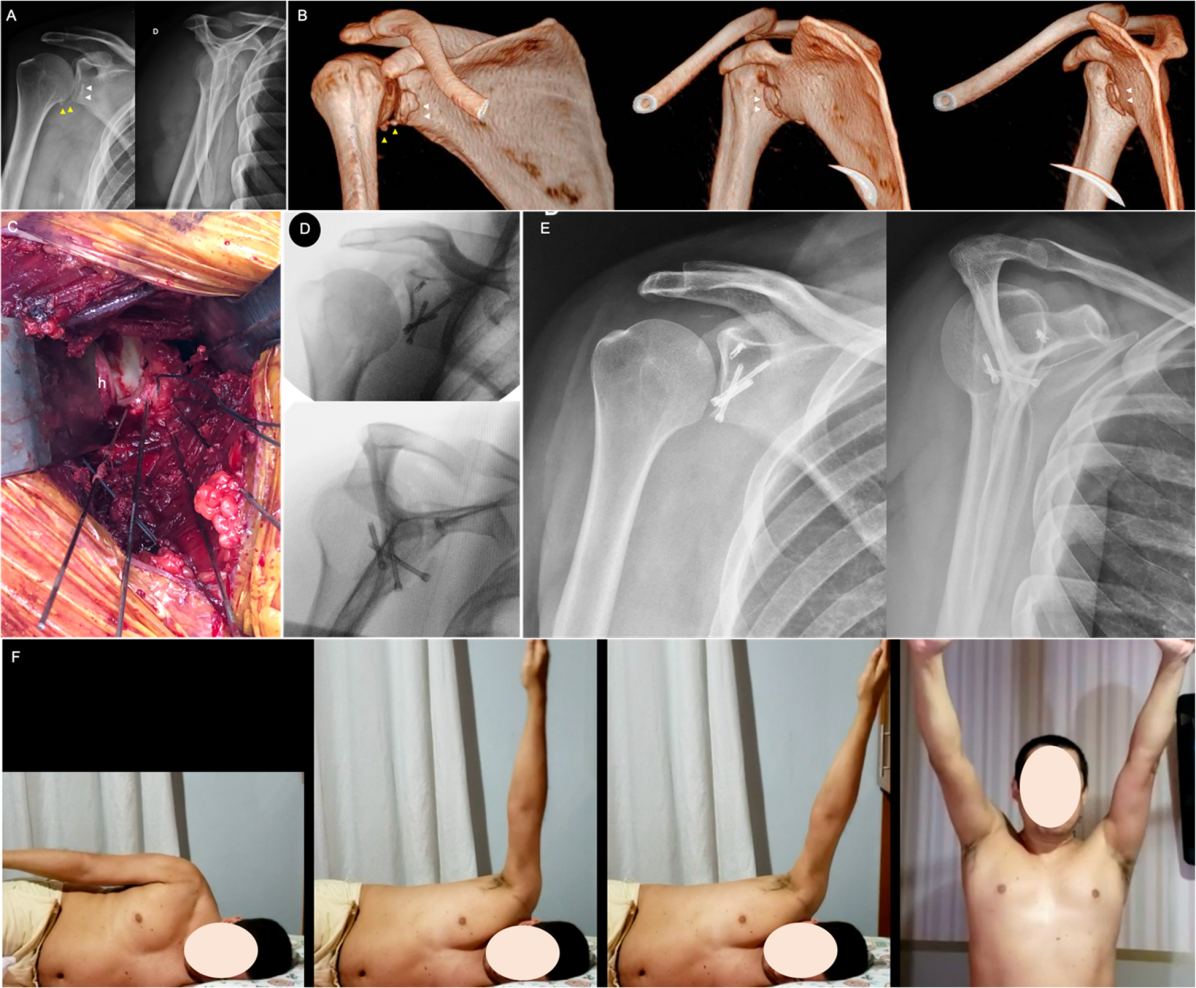
**a**: Preoperative true AP and lateral scapular radiographic views of the right shoulder of a 40-years-old male patient, showing a step-off on the anteroinferior rim of the glenoid (white arrowheads). Patient reported on a fall from stairs 48 h before. Also note the small bone fragments in the inferior portion of the capsule (yellow arrowheads); **b**: Preoperative 3-D CT reconstructions showing the displaced anteroinferior glenoid rim fracture (white arrowheads) and small bone fragments in the inferior portion of the capsule (yellow arrowheads); **c**: Intraoperative image showing the anteroinferior rim fracture anatomically reduced and provisionally fixed with multiple threaded K-wires. Observe the number 2 ethibond® sutures attached to the anterior labrum for posterior repair. * – anteroinferior glenoid rim fragment, h – humerus head; **d**: Intraoperative true AP and lateral scapular fluoroscopic views of the right shoulder showing final fixation with three 2.4-mm headless screws. Labrum was repaired using a bone anchor and unabsorbable sutures; **e**: Postoperative true anteroposterior and lateral scapular radiographic views of the right shoulder demonstrating the anatomic reduction of the anterior glenoid rim; **f** Pictures done during the rehabilitation protocol, demonstrating a satisfactory range of motion of the operated shoulder

We prefer an anterior approach for anterior fracture types carrying > 20% of the glenoid fossa and avulsed anteroinferior glenoid rim fractures overhanging the scapular neck more markedly than other parts of the glenoid fossa. Patient is placed in a 30° beach-chair position and operated on a complete radiolucent Table. C-arm imaging is checked before beginning the operative procedure. The anterior axillary incision described by Leslie and Ryan [47] is preferred to approach the anterior glenoid cavity. We normally inject between 20 and 30 mL of 2% lidocaine with adrenaline at 1:200,000 into the incision site to reduce bleeding during initial dissection. After skin incision, superficial dissection is done through the deltopectoral interval. The conjoint tendon is retracted medially using a blunt asymmetric Sofield retractor and the subscapularis tendon is opened to allow capsule exposure. Articular joint is finally exposed and a Fukuda retractor is positioned to lateralize the humerus head. The fracture is reduced under direct visualization and provisionally fixed with 1.0- or 1.2-mm smooth K-wires. We prefer to definitively fix the fracture with 2.0-mm headless cannulated screws or 2.0-mm cortical screws sinking the head to avoid damage to the humeral head. Labrum is frequently ruptured and must be sutured and reinserted using 1.5- or 2.0-mm anchors (Fig. 5).

**Fig. 6:**
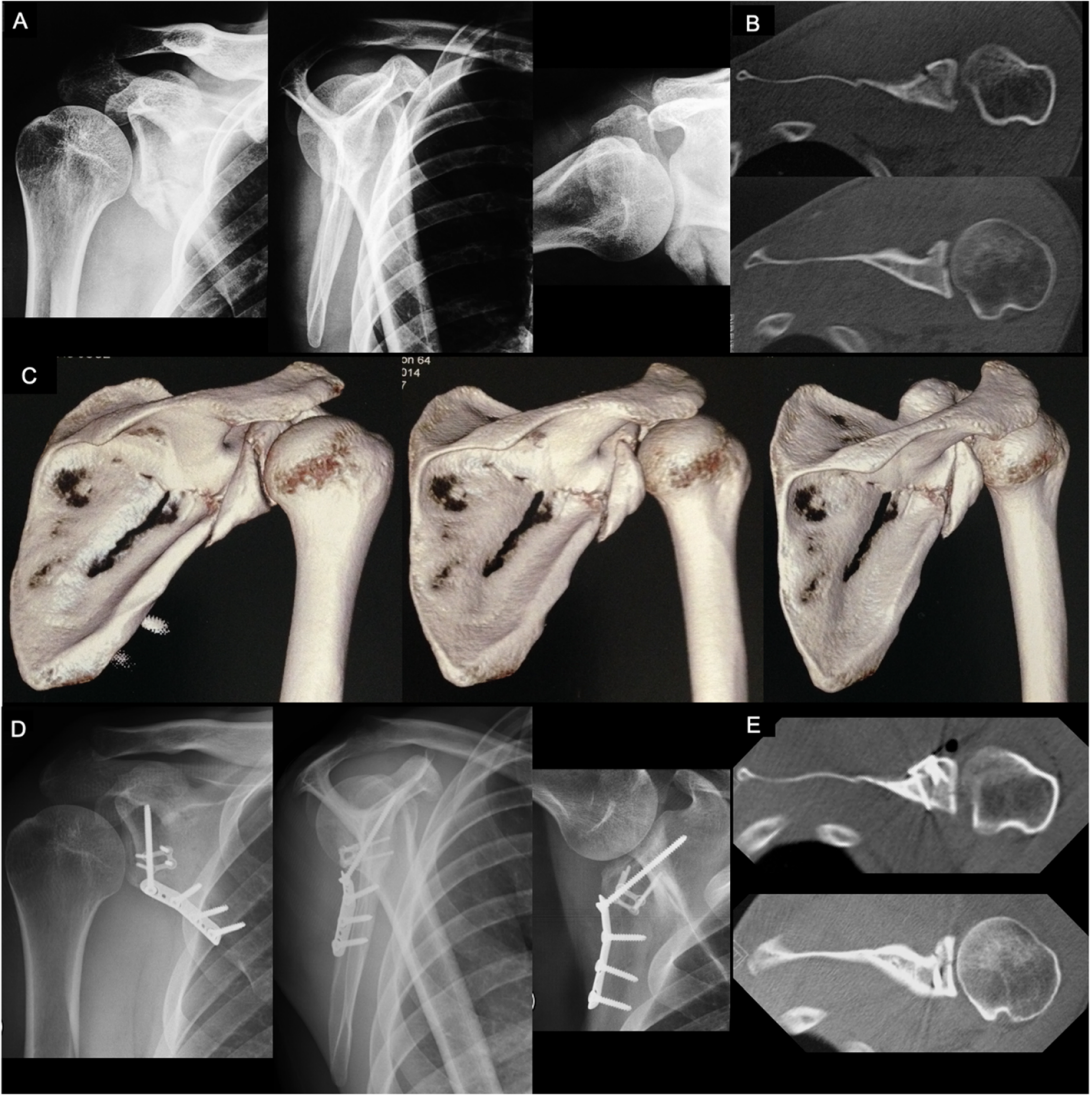
**a**: Preoperative true AP, lateral scapular, and axillary radiographic views of the right shoulder of a 25-years-old male patient, showing a displaced inferior glenoid fragment extending to the lateral pillar of the scapular neck and body. Patient reported on a fall from stairs 48 h before. Also note the small bone fragments in the inferior portion of the capsule (yellow arrowheads); **b**: Preoperative CT axial cuts of the right shoulder demonstrating the displaced inferior glenoid fracture; **c**: Preoperative 3-D CT reconstructions showing the displaced inferior glenoid fracture extending to the lateral pillar of the scapular neck and body; **d**: Postoperative true AP, lateral scapular, and axillary radiographic views of the right shoulder demonstrating the anatomic reduction of the inferior glenoid fracture and buttressing with a one-third tubular plate. Observe the 2.3-mm reduction plate used to maintain the reduction during surgery. Note the long 3.5-mm screw inserted through the plate directed to the coracoid process; **e**: Postoperative CT axial cuts of the right shoulder demonstrating the anatomic reduction of the inferior glenoid fracture

We prefer to use posterior approaches for posterior rim fractures carrying > 25% of the glenoid fossa and for all other glenoid fossa fracture patterns. Nowadays combined limited or minimally invasive approaches are preferable and were described previously in this review [31, 32]. For isolated posterior rim fractures we prefer the Brodsky [31] straight simplified longitudinal approach. For all other types involving a main fracture line running across the scapula into its medial border, we prefer the small surgical windows described by Gauger and Cole [32]. As stated before, the medial component of the fracture must be reduced and fixed with a relatively flexible implant first as it acts as a hinge to allow better manipulation, reduction, and final fixation of the lateral component [48]. We prefer locked or non-locked 2.0- and/or 2.3-mm straight plates located over the medial ridge of the scapular body. For the lateral component, we generally use miniplates as reduction tools before applying a non-locked one-third tubular plate buttressing the inferior glenoid neck and fossa. A long 3.5-mm cortical screw is inserted through the plate directed to the coracoid process (Fig. 6) [26, 34, 39] .

Postoperative radiographs and CT scan are used to assess both the quality of reduction and inadvertent articular penetration. Adequate pain control is mandatory to allow the beginning of the postoperative rehabilitation protocol. When the pain is reasonable under control (Visual Analogue Scale (VAS) between 2 and 3), both passive and active exercises are stimulated to regain progressive range of motion and proprioception of the operated shoulder. Also, patients are told to exercise the ipsilateral elbow, wrist, and fingers. Patients are advised to avoid heavy objects with the operated upper limb during a minimum of six weeks after the operation. Progressive strengthening is started after this period until bone healing.

#### Complex fractures of the scapula

Based on the study by Bartoníček et al. [46], we define complex fractures of the scapula when there is an entire glenoid fracture combined with a comminuted fracture of the whole scapular body. We noticed that in this high-energy fracture morphology, patients present an elevated number of thoracic injuries, such as multiple rib fractures with or without flail chest and pulmonary contusion with haemopneumothorax. Also, abdominal blunt injuries and cervical spine injuries have been observed in these patients, potentially leading to increased risk of complications and fatal outcome. Veysi et al. [49] carried out a retrospective review of 1164 multiple injured patients, defined as an Injury Severity Score (ISS) > 16, with chest and musculoskeletal injuries. In this group, 79 (6.8%) patients sustained a scapula fracture. They observed a significantly higher overall ISS in the group of patients with scapula fractures, with a significantly higher incidence of rib fractures. These patients also showed more severe chest injuries, although this finding did not raise statistically significance. The incidence and severity of head and abdominal injuries, and the rate of admission, the length of intensive care unit stay, and the overall length of hospital stay groups were similar between patients with and without scapula fractures. The severity of extremity injuries in patients with scapula fractures was significantly lower than in those without scapula fractures.

**Fig. 7:**
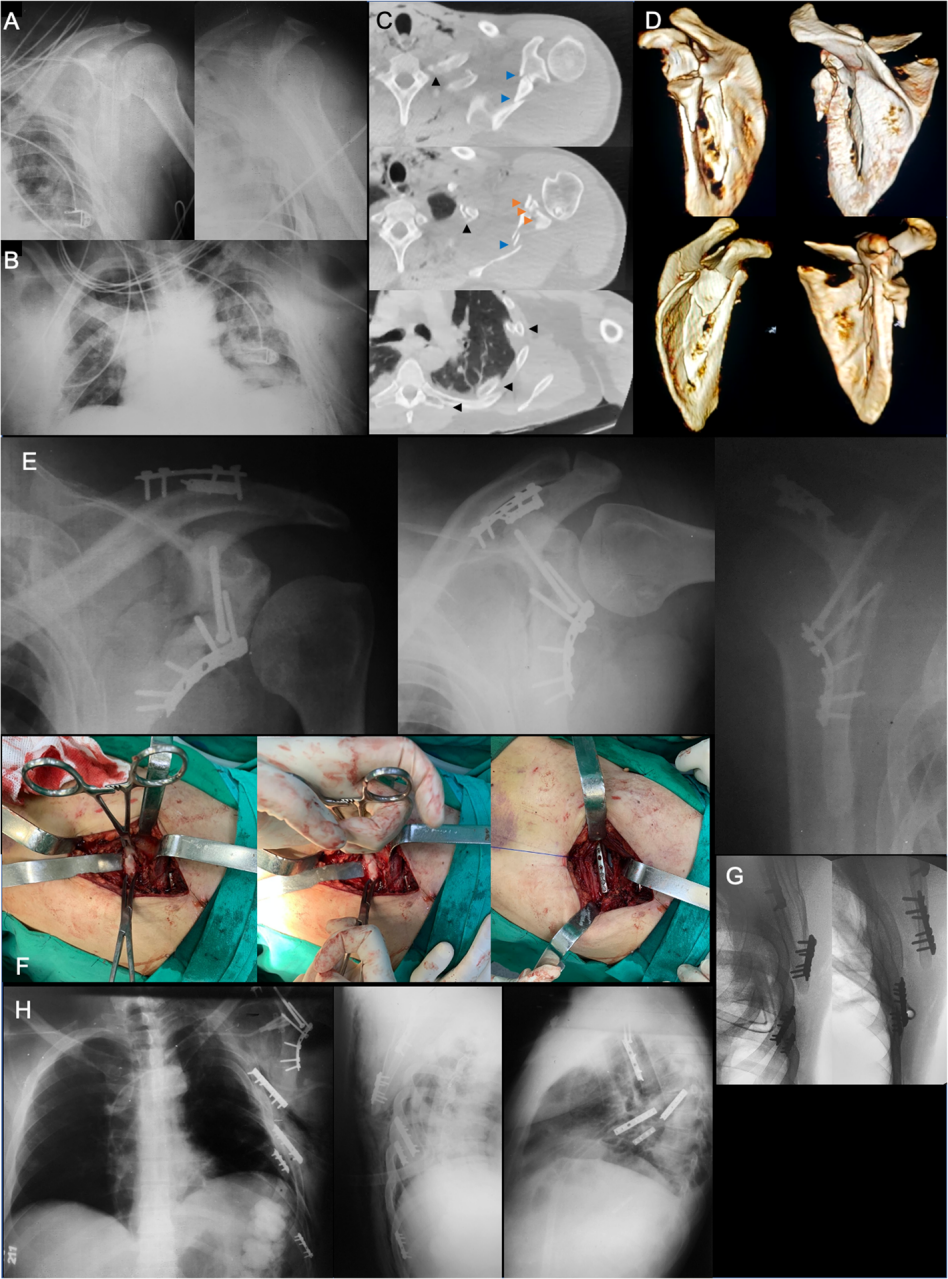
**a**: Preoperative AP and lateral scapular radiographic views of the left shoulder of a 42-years-old male polytraumatized patient done in the Intensive Care Unit (ICU), showing a comminuted displaced complex scapula fracture; **b**: Anteroposterior (AP) radiograph of the thorax done in the ICU, demonstrating a drain tube in the left hemithorax due to a traumatic haemopneumothorax; **c**: Preoperative CT axial cuts of the left shoulder and hemithorax, demonstrating the comminuted displaced complex scapula fracture, involving fragmentation of the glenoid fossa (orange arrowheads) and the scapular body (blue arrowheads). Note the multiple contiguous displaced rib fractures (black arrowheads), extending from the 3rd to the 9th left rib; **d**: Preoperative 3-D CT reconstructions showing comminuted displaced complex scapula fracture, involving fragmentation of the glenoid fossa and the scapular body. Observe the angled fracture of the spine of the scapular; **e**: Immediate postoperative true AP, AP, and lateral scapular views of the left shoulder demonstrating the fixation of the most proximal fractures of the scapula. Note the anatomic reduction of the glenoid fossa fracture. Patient was operated on in two steps, apart 5 days from each other; **f**: Intraoperative images of the 2nd operative procedure performed for the management of some rib fractures and the inferior angle of the scapular body. Observe the sequential reduction and fixation of the 6th left rib with a 2.0-mm straight non-locked plate; **g**: Intraoperative fluoroscopic images demonstrating the final fixation of the 6th, 7th, and 9th rib fractures, and the inferior angle of the scapular body; **h**, Postoperative AP, oblique, and lateral radiographs of the thorax, demonstrating the adequate reduction of both the complex left scapular and the multiple left rib fractures. Postoperative in-hospital and after discharge management protocols are the same as previously described for glenoid cavity fractures

During initial management, all life-threatening injuries should be rapidly identified and controlled, aiming to restore physiologic stability, avoid complications, and prevent further damage to the vital organs [50, 51]. In the stable patient, operative treatment is advisable to anatomically restore the glenoid cavity and adequately reconstruct both the scapular neck and body to allow a pain-free motion of the shoulder. Again, we prefer to use limited and/or minimally invasive approaches for acute fractures (Fig. 7), leaving more extensile approaches for delayed cases [28, 31, 32].

#### Fractures of the acromion

Acromion fractures are rare, accounting for 8 to 16% of all scapular fractures [9, 52, 53]. The classic injury mechanism is a direct impact over the lateral surface of the shoulder, usually occurring as a result of high-energy trauma. Although uncommon, acromion fractures can occur after acromioplasty, when the bone removed for the acromioplasty thins out the acromion to the point that it fractures secondary to forces applied to it by the upper extremity [54]. Iatrogenic acromion fractures are more common after arthroscopic surgery than after open acromioplasty [55]. Moreover, acromion fractures may also occur as a postoperative complication following reverse shoulder arthroplasty due to changes in shoulder length and biomechanics [18]. Associated fractures in other regions of the scapula, as well as clavicle and rib fractures, pulmonary contusion, and brachial plexus injury may also be part of the acromion fracture scenario.

Diagnosis is sometimes difficult and a high-level of suspicion should be raised in the patient complaining of severe shoulder pain after a direct trauma to the lateral aspect of the shoulder. Although the best radiograph for detection is an axillary view, these fractures can be difficult to see with conventional radiography. Therefore, a CT scanning or sometimes a Magnetic Resonance Image (MRI) may be necessary to better visualize the fracture [54]. The main differential diagnosis is the os acromiale, which is present in approximately 3% of the population [56, 57].

As previously mentioned, our preferred classification for acromion fractures is the Kuhn et al. [19] system, which takes into account the existence of displacement and reduction of the subacromial space. Unfortunately, there is no consensus regarding the treatment of acromion fractures. The singular, complex, and thin anatomy of the acromion, as well as its multiple ligamentous and muscular attachments make the treatment challenging. Conservative treatment is indicated for undisplaced, stable fractures (Kuhn et al. type-I) and must be thoroughly monitored due to the risk of progressive fracture displacement, especially in the type-IB. However, factors such as the length of immobilization, initiation of active motion and the type of orthosis are not consistently addressed in the literature. Kuhn et al. [19] recommended a simple sling for two weeks in cases of Type-IA fracture and for 4 to 12 weeks in the type-IB fracture. On the other hand, Hess et al. [18] recommended the use of a sling for 6 weeks with passive mobilization starting after 3 weeks and sling removal and active motion after 6 weeks. Ringelberg [58] demonstrated that the average force generated by the middle third of the deltoid to maintain the arm at 45 degrees of abduction is higher than 400 N. The author concluded that there is a considered deltoid traction over the acromion, even with no resistance during shoulder movement. Anecdotally, although it is believed that the use of an abduction sling can be more effective to reduce the lever arm of the deltoid muscle and prevent secondary displacement, nothing has been reported in the literature [18]. Symptomatic nonunion is one of the most frequently occurring complications following conservative treatment and has been associated with persistent pain, rotator cuff tears secondary to subacromial impingement, subluxation of the humeral head, shoulder weakness, and reduced function of the shoulder [59]. It has been suggested that nonsurgical treatment of an undisplaced or minimally displaced acromion fracture associated to SSSC disruption presents a higher risk of failure due to instability of the shoulder girdle and secondary displacement of the fracture [37].

There are no absolute indications for surgical treatment of acromion fractures. However, Gorczyca et al. [60] reported that, although conservative treatment of displaced fractures of the acromion may result in satisfactory function, measurement of shoulder strength after non-operative treatment of the displaced fractures of the acromion has yet to be reported. Indeed, several complications have been associated with non-operative management of displaced acromion fractures, including pain, decreased range of motion, rotator cuff tears secondary to subacromial impact, subluxation of the humeral head, shoulder weakness, and symptomatic nonunion [61–64]. Hardegger et al. [9] recommended osteosynthesis for acromion fractures with significant displacement to prevent painful nonunion and to protect the rotator cuff from subacromial impingement. We believe that conservative treatment of displaced acromion fractures could result in weakness of the deltoid muscle, therefore strongly impairing the shoulder girdle function. Therefore, we indicate surgery for all displaced Kuhn et al. type-II and type-III acromion fractures.

**Fig. 8:**
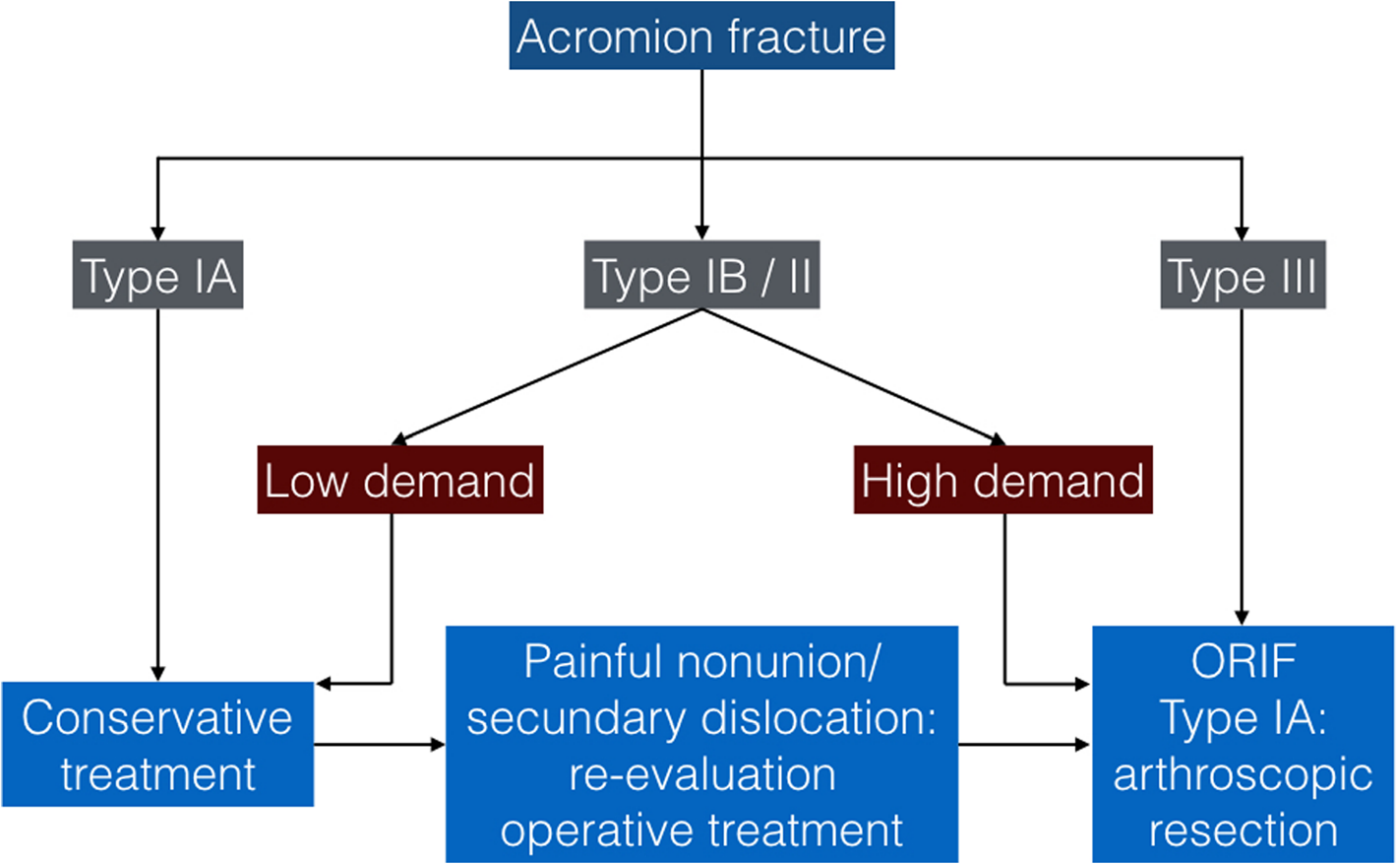
Proposed treatment algorithm for acromion fractures by Hess et al. [18] The classification is based on the original system described by Kuhn et al. [19]

Several fixation options have been described for the treatment of acromion fractures, including K-wires only or as part of a tension band technique, screws only, plates and screws especially designed for acromion fractures, or unconventional customized plates with or without arthroscopy aid [58, 60, 62, 65]. Kim et al. [63] compared early with delayed fixation of acromion fractures in a retrospective series of 34 patients and found a significantly better Constant Score on the early fixation group. Hess et al. [18] drafted a helpful treatment algorithm based on the Kuhn et al. [19] classification. The treatment strategy is focused on both initial fracture displacement and patient physical demand. Patients presenting displaced fractures who are physically active, employed, and living independently are typically assigned to the high demand group, regardless of their age. Figure 8 depicts the Hess et al. [18] algorithm, which also represents our rationale for treatment.

**Fig. 9:**
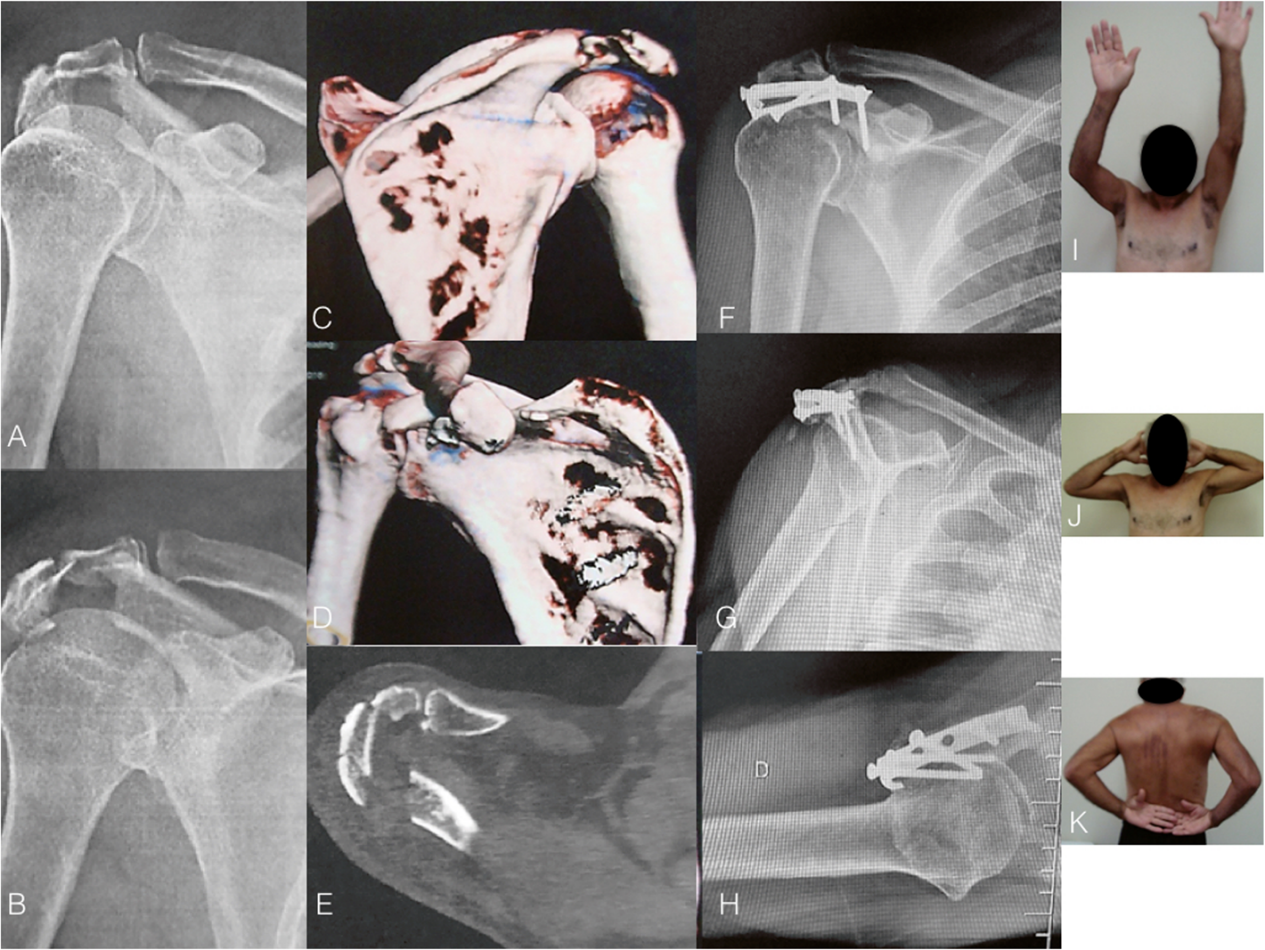
**a** and **b**: Radiographs of the shoulder showing a Kuhn et al. [18] type-II multifragmentary fracture of the acromion extending to the most lateral part of the scapular spine. **c**, **d**, and **e**: Observe the amount of comminution on the CT scan. There is no obvious reduction of the subacromial space. **f**, **g**, and **h**: Fracture fixation was performed with a superiorly placed non-locked one-third tubular plate. **i**, **j**, and **k**: Observe the functional range of motion of the operated shoulder after fracture healing at 24 months postoperatively

Our standard approach for displaced acromion fractures is the posterior straight skin incision with extension to the scapular spine. If posterior glenoid fossa, glenoid neck, or scapula body fractures are associated with an acromion fracture, the Brodsky [31] approach with proximal curved extension is a helpful alternative. Our fixation strategy usually combines stronger plates with minifragment implants. We normally prefer to associate 3.5- and/or 2.8-mm conventional or locking plates with a minifragment 2.4-mm plate, depending on the fracture pattern. Distal radius plates may be unconventionally used, particularly when there is an extension of the fracture into the scapular spine. If an oblique fracture line is present in the absence of comminution, lag screws can be placed, usually outside the plate. Additional sutures can be placed to increase fixation stability. However, careful soft tissues dissection must be performed to prevent devitalization around the fracture site. Also, in the very skinny patient, it is preferable to use minifragment implants to avoid hardware protrusion, soft tissue discomfort, and wound complications. Figure 9 shows an acromion fracture fixation.

#### Fractures of the coracoid process

The coracoid process is part of the superior shoulder suspensory complex and contributes to the anterosuperior stability of the glenohumeral joint [36, 37, 66]. A fracture of the coracoid process is a rare injury, with McGinnis and Denton [67] describing the prevalence of coracoid fractures between 3 and 13% of all scapula fractures. Data from two systematic reviews of scapular fractures in 2006 and 2008 reported the prevalence of apophyseal (acromion, coracoid, and scapular spine) fractures to be up to 8.2% [22, 53]. Fractures of the coracoid process are typically caused by high-energy trauma and are often seen in combination with other injuries [16, 17, 68]. The vast majority of case reports and series of coracoid fractures is associated with concurrent shoulder injuries, most commonly located at the acromioclavicular joint [69].

Isolated coracoid fractures that are either nondisplaced or minimally displaced can be successfully treated with nonsurgical management [70–72]. Even displaced isolated coracoid tip and fractures located between the coracoclavicular and coracoacromial ligaments can be successfully treated with nonsurgical management [73, 74]. Indications for surgical treatment include more than 10 mm of displacement, multiple disruptions of the SSSC, and symptomatic nonunions [73, 74]. Coracoid process fractures may also be displaced by the traction of the short head of the biceps tendon, thereby requiring surgical treatment depending on the amount of displacement.

For coracoid process fracture fixation, the patient is placed in the beach chair position on a radiolucent table. The C-arm can be positioned either on the opposite side or behind the shoulder to allow for at least 2 orthogonal views. Bathia [75, 76] suggest the image intensifier to be positioned in the anteroposterior plane, so fluoroscopic versions of two specialized radiographic coracoid pillar views can be done to visualize two coracoid pillars. The cephalad and lateral angulations (30 to 40° each) of the fluoroscopic beam directed at the coracoid tip demonstrates the entire profile of the superior coracoid pillar (‘superior pillar view’) and the cephalad and medial angulations (30 to 40° each) of the fluoroscopic beam demonstrates the entire ‘inferior coracoid pillar’. van Trikt et al. [77] described the coracoid tunnel view based on simple landmarks of the scapular bone. They found the optimal passageway of a screw through the coracoid base into the neck of the scapula as the coracoid tunnel. Starting with the anteroposterior fluoroscopic view, the glenoid fossa, coracoid, acromion, scapular notch, superior scapular border, medial border, inferior border, and scapula spine are identified. Then, the fluoroscopic beam is moved in a cephalad direction until an oval shaped tunnel (the coracoid tunnel) is projected between the coracoid tip, glenoid fossa, scapular notch, and superior scapular border. Finally, the beam is re-adjusted until the glenoid fossa is parallel to the drilling direction, making sure that the superior border of the scapula is kept into roughly one line.

**Fig. 10:**
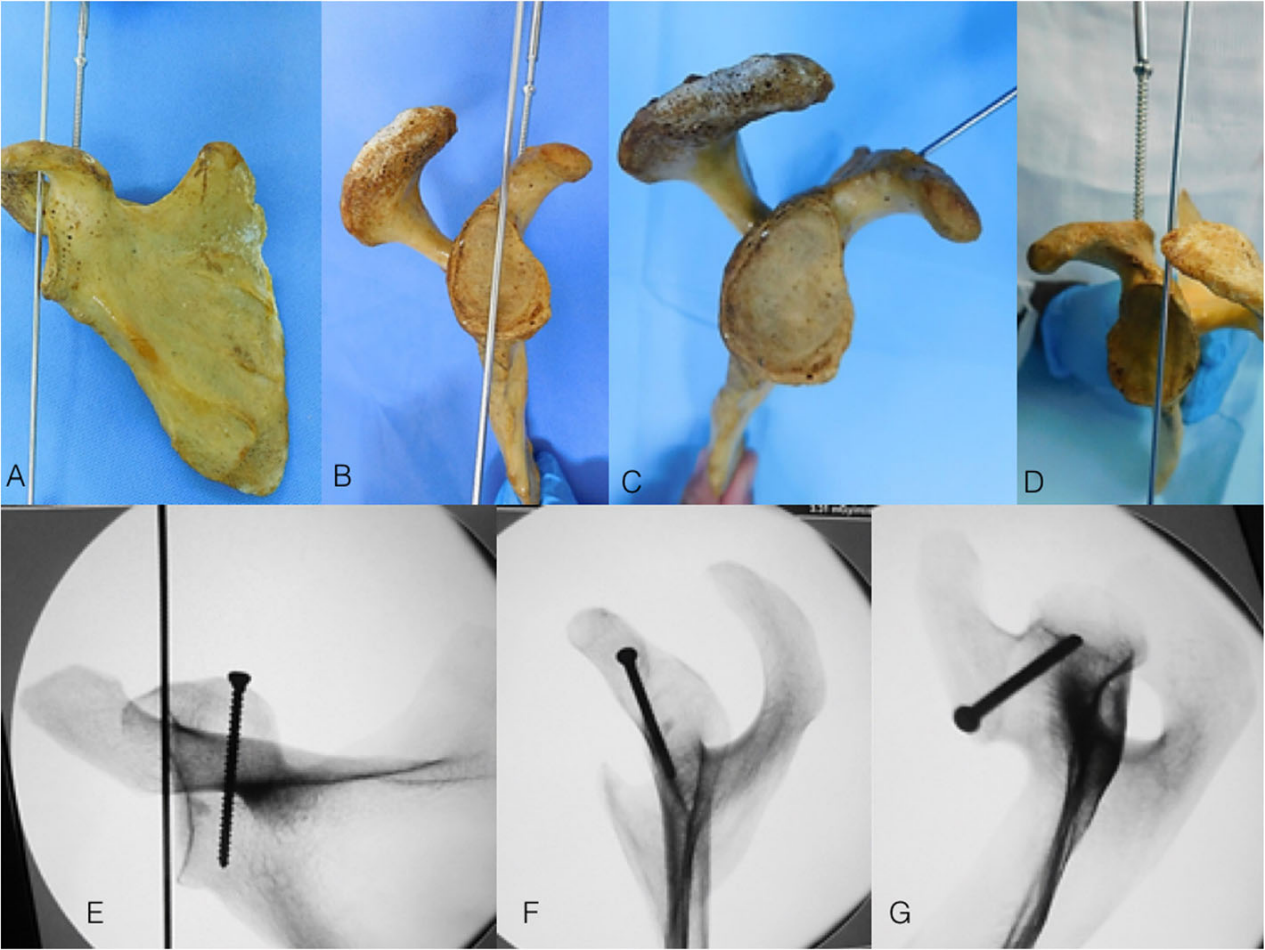
**a**, **b**, **c**, and **d**: Photographs of a scapular specimens showing the placement of K-wire parallel to the glenoid fossa, guiding the screw placement into the coracoid process. Observe that the screw must be positioned parallel to the longest axis of the glenoid. **e**, **f**, and **g**: Fluoroscopy images showing the coracoid fixation

Coracoid fractures can be addressed with an anterior deltopectoral Henry approach, although some nondisplaced Ogawa et al. [17] type-1 fractures can be fixed percutaneously [75, 76]. The arm is internally rotated and adducted to protect the brachial plexus. The entire limb should be prepped and draped to allow for intraoperative elbow flexion, which can relieve the traction force caused by the biceps tendon [73]. After fracture reduction, a 2.0-mm K-wire is used to temporarily maintain the reduction. A correct and accurate screw placement is essential to achieve adequate stability and prevent fixation failure [75–79]. As mentioned before, the sharp, hooked, and thin coracoid tip precludes the screw placement starting from this landmark. Therefore, the screw must be placed down the coracoid body through the coracoid base and into the neck of the scapula, which is the coracoid tunnel [77]. In the vast majority of cases, the drill must be positioned perpendicular to the coracoid process and parallel to the longest axis of the glenoid cavity and the screw must be placed parallel to the glenoid fossa. Care must be taken so the screw does not violate or penetrate the osseous borders of the coracoid tunnel. We usually perform the fixation with a 3.5-mm cortical screw or less commonly with a partially threaded 3.5-mm cannulated screw. Although rare, in certain fracture patterns with very large coracoid base, a second screw may be necessary and/or a minifragment plate may additionally be used to increase construct stability. Figure 10 depicts the safe placement of the coracoid process screw through the coracoid tunnel.

**Fig. 11:**
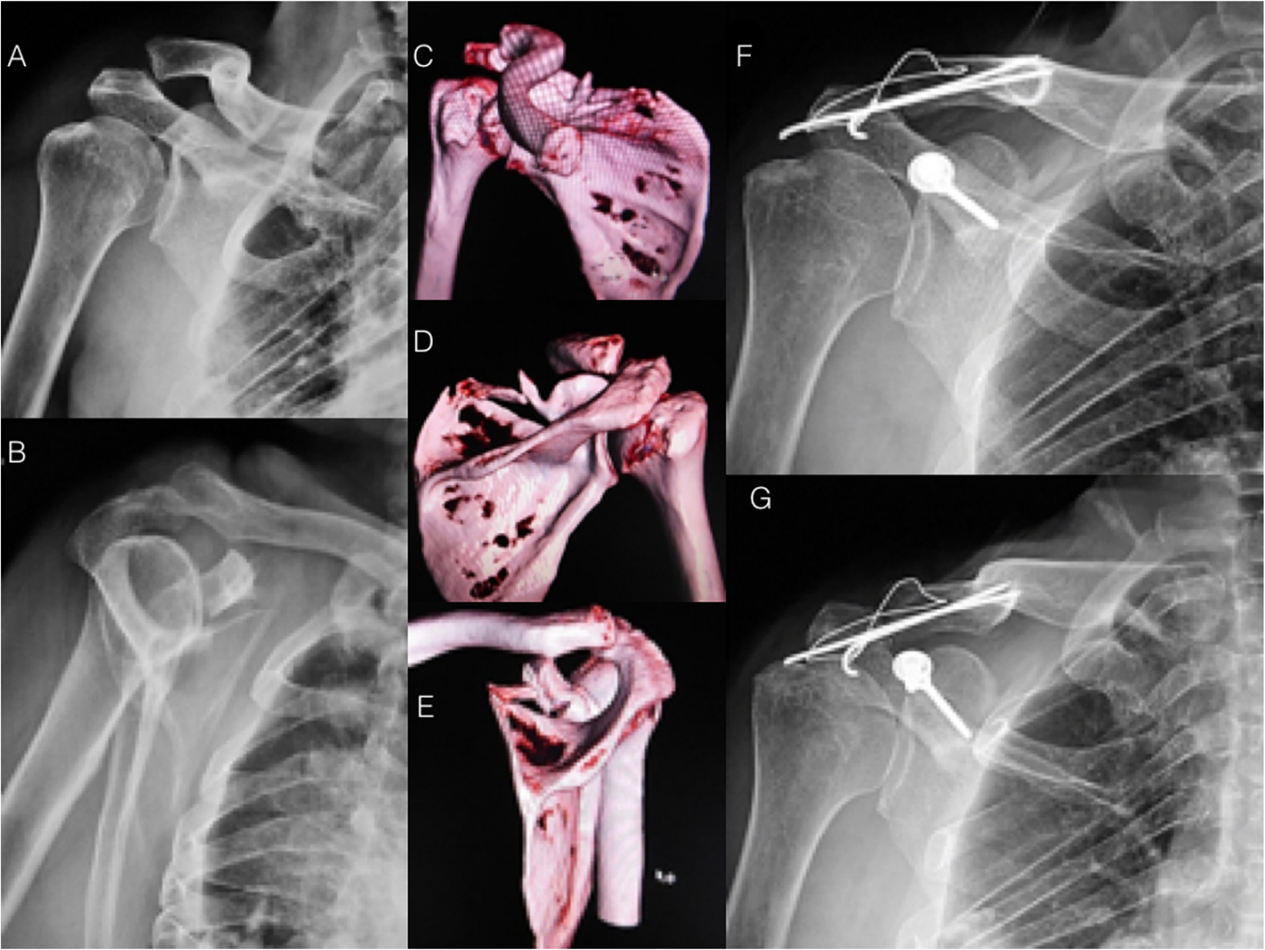
Radiographs (**a** and **b**) and 3D-CT reconstruction (**c**, **d**, and **e**) showing the fractures of the coracoid process and scapular spine associated with acromioclavicular dislocation. **f** and **g**: Postoperative radiographs showing the coracoid process fracture fixation with two 3.5 mm cortical screws and the acromioclavicular fixation with a static tension band

Figure 11 shows a case of coracoid process fracture associated with acromioclavicular dislocation.

Ogawa et al. [17] retrospectively reviewed 67 patients with isolated coracoid fractures. Forty-five patients were available for a follow-up at a mean of 37 months (12 to 117 months). No notable difference was observed in the outcomes between patients with type-1 and 2 fractures according to their classification between those undergoing conservative and surgical treatment. Hill et al. [73] analyzed the outcomes of 22 patients with isolated coracoid process fractures treated with surgical fixation. A total of 17 patients underwent open reduction and internal fixation with one to three lag screws, whereas five patients underwent surgical fixation with a combination of screws and minifragment plates. At a mean follow-up of 23.5 months, the median Disabilities of the Arm, Shoulder and Hand (DASH) score was 12.3 (range, 0 to 74; mean, 10.1) and 16 (84%) returned to previous work or employment. In our opinion, due to the rarity of this fracture type and the inconsistencies in results from existing studies in terms of surgical indications, the decision regarding the modality of treatment should be thoroughly shared with the patient for a correct and individualized management based on the fracture pattern, associated shoulder injuries, patient activity level, and patient’s expectation.

## Conclusion

Treatment of scapular fractures remains challenging. Although the vast majority of scapula fractures may be safely managed with conservative treatment, caution should be taken to not miss the opportunity to correctly indicate the surgical treatment in selected cases. The anterior axillary incision described by Leslie and Ryan is preferred to approach anterior fracture types carrying > 20% of the glenoid fossa and avulsed anteroinferior glenoid rim fractures overhanging the scapular neck more markedly than other parts of the glenoid fossa. The modified Judet approach preserving the posterior deltoid attachment is a helpful and effective approach to fix posteriorly displaced scapula fractures. Limited and/or minimally invasive approaches represent an interesting alterative for some posteriorly displaced fractures, with the potential advantage of an early rehabilitation protocol, but with the drawback of requiring a long learning curve in scapula fractures fixation. Finally, with a better understanding of the indications for surgical treatment and with the soft-tissue-preserving procedures, satisfactory functional outcomes could be achieved, with low complication rates.

## Abbreviations

CT: Computed tomography
3D: Three dimensional
ORIF: Open reduction and internal fixation
SSSC: superior shoulder suspensory complex
GP: Glenopolar
VAS: Visual Analogue Scale
AO/OTA: Arbeitsgemeinschaft für Osteosynthesefragen / Orthopaedic Trauma Association
ISS: Injury Severity Score
ICU: Intensive Care Unit
MRI: Magnetic Resonance Image
DASH: Disabilities of the arm, shoulder and hand
